# Anesthesia for a parturient with intraneural perineurioma: A case report

**DOI:** 10.1097/MD.0000000000009135

**Published:** 2017-12-08

**Authors:** Jiao Li, Hong Zeng, Zhengqian Li, Jun Wang

**Affiliations:** Department of Anesthesiology, Peking University Third Hospital, Beijing, P.R. China.

**Keywords:** cesarean section, general anesthesia, intraneural perineurioma, neuraxial anesthesia

## Abstract

**Rationale:**

Intraneural perineurioma is an extremely rare form of peripheral nerve sheath tumor; and the anesthetic management of a parturient with intraneural perineurioma, especially affecting spinal roots and nerves of extremities, is very rare.

**Patient concerns:**

A 28-year-old woman was referred to the hospital at 37+5 weeks’ gestation, presenting with a 10-year history of paroxysmal acroanesthesia and aching with distal limbs.

**Diagnoses:**

Based on the biopsy results, including immunohistochemical and electron microscopic findings, and molecular studies, her condition was diagnosed as intraneural perineurioma.

**Interventions:**

The size of pelvic nervous masses gradually increased with pregnancy. A scheduled elective cesarean section under general anesthesia was concluded for the patient under preoperative multidisciplinary consultation with anesthesiologist, neonatologist, and neurologist.

**Outcomes:**

The patient and the neonate were discharged smoothly on the fourth postoperative day. During a 6-month follow-up period, no new neurologic complication was observed.

**Lessons:**

To our knowledge, this is the first case report that documented the anesthetic management for a parturient with intraneural perineuroma. Careful preconception care and multidisciplinary assessment are essential to achieve optimal reproductive outcomes.

## 1 Introduction

Perineurioma, a benign peripheral nerve sheath neoplasm, was first histologically identified by Imaginario in 1964.^[1]^ The tumor cells in perineurioma are characterized by their specific immunohistochemical and ultrastructural features.^[2]^ According to the location, perineurioma can be divided into 2 forms: intraneural and extraneural (soft tissue) perineurioma. To date, approximately 120 cases with intraneural perineurioma have been published.^[3]^ Nevertheless, the anesthetic management for a parturient with intraneural perineurioma has not been reported. As the spinal roots and nerves of extremities are involved, neuraxial anesthesia should be theoretically avoided. Therefore, general anesthesia may be a safer option.

## 2 Methods

We report an observational case of intraneural perineurioma. The committee waived the requirement for approval to conduct this single case study with access to medical records. Informed consent to publication has been obtained from the patient.

## 3 Case report

A 28-year-old woman G1P0 (70 kg, 160 cm) was admitted to hospital at 37+5 weeks’ gestation. She was originally confirmed the diagnosis of an intraneural perineurioma at the age of 22 years. At that time, she presented with a 10-year history of paroxysmal acroanesthesia primarily localized in distal limbs and accompanied with aching. Nerve conduction studies demonstrated velocity was reduced in bilateral median, ulnar, radial, tibial, and sural nerves. Magnetic resonance imaging (MRI), especially T2-weighted images, demonstrated enlargement of the nerves, including the cervical plexus, brachial plexus, intercostal nerves, spinal roots, sciatic nerve, and so on. Her histopathological examination of a cross section of the right sural nerve showed expanded nerve fascicles composed of spindle-shaped perineurial cells around a central axon, arranged in pseudo-onion bulbs. Immunohistochemical staining showed that the perineural cells were positive for epithelial membrane antigen (EMA), collagen IV, and CD34, and that the Schwann cells were positive for S-100. Electron microscopy findings also demonstrated concentric arrangements of perineurial cells around a central axon, accompanying with Schwann cell. All of these evidences were consistent and indicated intraneural perineurioma. Fluorescene in situ hybridization (FISH) analysis was further performed to explore the genetic mechanisms and to differentiate with other hereditary neuropathies. In our case, the deletion of locus of chromosome 22 (22q11) was negative and no deletion on 17p11.2-p12, excluding the possibility of HNPP (hereditary neuropathy with liability to pressure palsy) and no mutations in exon2 or exon3 of Neurofibromatosis type 2 (NF-2). Genetic counseling recommended screening tests for hereditary sensory and motor neuropathy (HMSN), hereditary spastic paraplegia (HSP), and amyotrophic lateral sclerosis (ALS). They were all negative. There was no family history of perineurioma.

**Figure 1:**
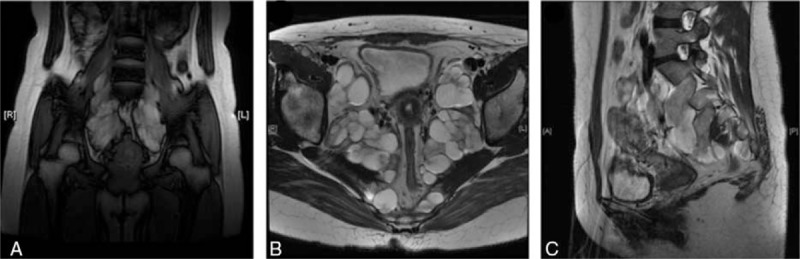
MRI demonstrated enlargement of lumbosacral nerves and abnormal signal on T2-weighted images in (A) the coronal, (B) the horizontal, (C) and the sagittal planes.

**Figure 2:**
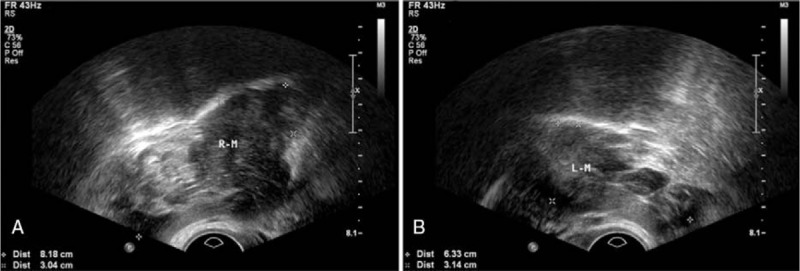
Preconceptional transvaginal ultrasonography (A) detected a heterogeneous and hypoechoic mass behind the right adnex (approximately 8.1 × 3.0 in size) and (B) left adnex (approximately 6.3 × 3.1 cm in size).

The patient was referred to the obstetric clinic for preconception counseling, when she was found to have bilateral enlarged lumbosacral nerves on MRI of pelvis (Fig. 1). At the mean time, results from transvaginal ultrasound scan revealed an increase in heterogeneous and hypoechoic mass (8.1 × 3.0 cm behind the right adnex and 6.3 × 3.1 cm behind the left; Fig. 2). Obstetrician and neurologist suggested attention to the possibility of rapid increase in the size of pelvic nervous masses and exacerbation of symptoms during pregnancy. One month later, she was pregnant and solid masses were palpated behind uterus on bimanual examination, with numbness and electric shock feeling of lower limbs. Even though she denied any changes in her neurological symptoms throughout the whole pregnancy, follow-up ultrasound studies were performed regularly. The size of pelvic nervous masses was depicted in Table 1, including before, during pregnancy, and postpartum.

**Table 1:**
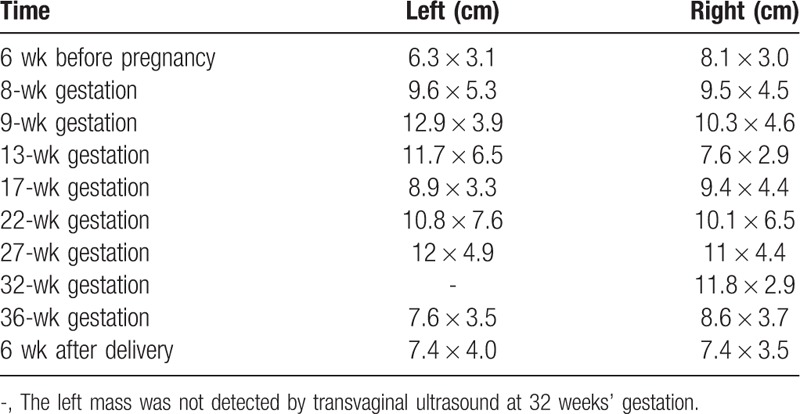
The size of pelvic masses on the transvaginal ultrasound at different times.

There was a concern that natural or induced labor should be discouraged due to nerve compression during pushing in the second stage of labor. In addition, given the obvious neurological signs and the involvement of spinal roots and lumbosacral nerves indicated by MRI, any neuraxial technique could potentially be harmful to nervous system. After multidisciplinary consultation with anesthesiologist, neonatologist, and neurologist, it was considered that a scheduled elective cesarean section under general anesthesia was the safest option for the patient.

The patient underwent cesarean section at 38 weeks’ gestation. There was no change in her neurological status and the fetal heart rate was stable and reassuring before the surgery. A general anesthetic with rapid sequence induction was performed, when sterilization had been finished. First, propfol (2 mg/kg) was given, and then endotracheal intubation was facilitated with i.v. rocuronium (0.6 mg/kg). General anesthesia was maintained with 1.5% sevoflurane combined with 2 L/min of nitrous oxide, carried by 100% oxygen of 1.5 L/min. Approximately 3 minutes after the skin incision, a male neonate (weight, 3.5 kg) was delivered with Apgar scores of 10 at 1 minute and 10 at 5 minutes. No masses were found in innervational region of ulnar nerve of the neonate by neonatologist and neurologist. Two minutes after the delivery, the placenta was removed and intravenous infusion of 20 International Units (IU) of oxytocin mixed with 250 mL of 0.9% saline was commenced. Then an i.v. infusion of fentanyl (0.1 mg) was used to relieve postoperative analgesia and nitrous oxide was stopped for the preparation of recovery. The masses were touched behind the uterus during intraoperative pelvic exploration, but no further treatment was provided considering the pelvic engorgement during perinatal period. At the end of the surgery, the endotracheal tube was removed and the mother was smoothly recovered without any new neurological symptom or anesthetic complication. The neonate was transferred to the neonatal ward immediately for meticulous nursing. During the neonatal ward stay, he showed no abnormality and was discharged on the fourth postoperative day with his mother. At follow-up, half a year later, the patient revealed without new neurologic complaints.

## 4 Discussion

Perineuriomas are benign peripheral nerve sheath neoplasms composed of perineurial cells.^[4]^ They have been traditionally classified into 2 main types according to their location—intraneural and extraneural—and overlap histologically with many other tumors. Intraneural perineurioma is composed exclusively of perineurial cells restricted to the boundaries of a nerve and this term was proposed by Emory et al^[5]^ to combine lesions previously classified as localized hypertrophic neuropathy, hypertrophic mononeuropathy, localized hypertrophic neurofibrosis, intraneural neurofibroma, and hypertrophic interstitial neuritis. The typical age of onset of intraneural perineurioma is adolescence or young adulthood without sexual predilection. It primarily affects the extremities with associated motor deficiency and occasional sensory loss. The process is usually limited to a single major nerve. It has rarely been described in multiple nerves like our case. The nature of intraneural perineurioma was still a subject of debate.^[6,7]^ The literatures suggest that the long arm of chromosome 22 contains a tumor suppressor gene involved in the pathogenesis of nerve sheath tumors. The finding of deletion of 22q11-qter is of particular interest in that same clonal chromosome abnormality has been described in benign and malignant schwannomas,^[8]^ neurofibromas,^[9]^ meningiomas,^[10]^ and gliomas.^[11]^ From Emory's^[5]^ cytergenetic data of case 5, the tumor cells appeared to be homozygously deficient for the region 22q11.2qter. But there was no loss of this lesion in the present case.

In western societies, preconception care is widely recognized as a way to optimize women's health and improving pregnancy outcomes. Genetic counseling programs are becoming an important part^[12]^ of preconception care. The goal of genetic counseling is to educate the woman and her partner to understand their specific levels of risk, risk reduction, and reproductive options.^[13]^ Certain patients may be at a higher risk for having a fetus with a genetic disorder. Screening and diagnostic tests were recommended before pregnancy to offer them accurate information. There was no relevant gene discovery in HMSN, HSP, and ALS. In addition, the hormonal effects of pregnancy upon the growth of skin neurofibromas^[14]^ and acoustic neuromas^[15]^ has been described. Similarly, one case was described^[16]^ in parturient with NF2 who experienced an exacerbation of symptoms presumably due to an increase in size of a left brachial plexus tumor. Therefore, it is likely to occur under the influence of high levels of progesterone, including worsening symptoms, a high incidence of fetal distress, and emergency cesarean delivery. Fortunately, in the present case pelvic masses truly grew up along with gestation, without changes of her neurological symptoms. And no abnormalities of fetus were detected by prenatal ultrasound screening during the whole pregnancy.

Although anesthetic management of patients with intraneural perineurioma has not been specifically addressed, surgical removal of intraneural perineurioma arising in the brachial plexus is reported under general anesthesia.^[17]^ We had no previous experience of managing intraneural perineurioma parturient and related literatures did not offer any meaningful practical advice.^[18–20]^ We managed this case based on multidisciplinary consultation for reasonable expectations and experiences of managing parturients with other preexisting neurologic conditions such as neurofibromas.

From the consideration of obstetricians, vaginal delivery was discouraged due to the potential neurologic injury from nerve compression against the pelvis by the fetal head. This risk may increase with nulliparity, long second stage of labor, cephalopelvic disproportion, nonvertex fetal presentations, and forceps-assisted delivery.^[21]^ The mechanism is likely related to stretch or compression injury to the lumbosacral plexus or lower extremity peripheral nerves. Compression of the neurovascular supply is another possible mechanism.^[20]^ Moreover, women often push during the second stage of labor in a lithotomy position with their thighs hyperflexed on the abdomen. This position may stretch nerves as they course from the pelvis to the lower extremities.

For anesthesiologists, the decision to avoid neuraxial anesthesia was guided by enlarged spinal roots and lumbosacral nerves that make it difficult to perform neuraxial anesthesia, In addition, neuraxial anesthesia may cause direct neurologic injury and secondary injury to nervous system due to the potential risk of bleeding from perineurioma. Moreover, the presence of a motor block may also contribute to an inability to easily move and reposition. She may not be able to recognize symptoms of impending nerve injury and thus fail to take action, such as shifting position to relieve the nerve compression. Lastly, the potential does exist for a weakened or patchy block due to thick nerve fibers. Traditionally, thin nerve fibers were believed to be more easily blocked than thick ones.

Most perineuriomas are localized and self-limited. But the treatment of intraneural perineurioma has been controversial. In an attempt to preserve nerve function, some authors advocate diagnostic biopsy, whereas others prefer the resection with neural grafting or end-to-end anastomosis.^[22]^ In our case, the masses were palpated during intraoperative pelvic exploration, but not given further intervention according to the neurologists because of pelvic engorgement during pregnancy.

In conclusion, the management of a parturient with intraneural perineuroma with careful preconception care and multidisciplinary assessment warranted the optimal reproductive outcomes. Early consultation with anesthesiologists, neurologists, and obstetricians allows to minimize the risks of damage due to the mode of delivery and anesthetic options. Cesarean section under general anesthesia may be the safest option for delivery in a parturient with intraneural perineurioma.
